# Cataract progression following lens-sparing pars plana vitrectomy for rhegmatogenous retinal detachment

**DOI:** 10.1038/s41598-022-26415-4

**Published:** 2022-12-21

**Authors:** Carlo Bellucci, Lucia Benatti, Maurizio Rossi, Salvatore Antonio Tedesco, Arturo Carta, Giacomo Calzetti, Stefano Gandolfi, Paolo Mora

**Affiliations:** 1 Ophthalmology Unit, University Hospital of Parma, Parma, Italy; 2 Department of Clinical and Experimental Medicine, University Hospital of Parma, Parma, Italy; 3 Institute of Molecular and Clinical Ophthalmology Basel, Basel, Switzerland; 4 Department of Ophthalmology, University Hospital of Basel, Basel, Switzerland

**Keywords:** Lens diseases, Retinal diseases

## Abstract

Lens-sparing pars plana vitrectomy (PPV) is often followed by cataract development. However, there have been few prospective studies evaluating the timing of cataract progression and potential associated factors. This was an observational study conducted at the Ophthalmology Unit of the University Hospital of Parma (Parma, Italy). Patients presenting with rhegmatogenous retinal detachment (RRD), who underwent PPV with preservation of the lens, were examined according to a scheduled follow-up (3, 6 and 12 months after PPV) and then preoperatively when cataract extraction surgery (CES) was indicated, or at the end of the study follow-up period (May 2021). The primary outcome was the interval between PPV and CES indication (based on predefined refractive criteria). A total of 36 eyes of 36 patients (mean age: 52 ± 10 years) were included in the study. Nineteen eyes (53%) were indicated for CES a median of 14.5 months (IQR: 12.0–24.8) after PPV. The nuclear and posterior subcapsular forms of cataract progressed significantly starting at 6 months after PPV. Older age at the time of PPV, silicone oil tamponade and RRD without macular involvement were significantly and independently associated with an earlier indication for CES. Patient age and the use of silicone oil tamponade must be taken into consideration when evaluating the risk of cataract development after PPV.

## Introduction

In the phakic eye, pars plana vitrectomy (PPV) often results in cataract formation^1,2^. The incidence of visually disturbing cataract, referring to the development of nuclear sclerotic (N) and posterior subcapsular (PSC) forms, varies widely^3,4^. Lens-sparing PPV is an option widely used in the management of some phakic rhegmatogenous retinal detachment (RRD). A recent study reported similar results, in terms of the retinal reattachment rate and recovery of vision, after PPV with preservation of the crystalline lens or combined with phacoemulsification^5^. Due to the potential for cataract progression and difficulty of performing phacoemulsification in vitrectomized eyes, identifying potentially general and technical prognostic factors is important to guide the treatment approach when PPV is required. These essentially refers to: the patient age; the risk of postoperative refractive errors, especially in macula-off cases; the iatrogenic anisometropia in myopic subjects; the removal of a largely healthy organ in cases of no/mild cataract with residual accommodative function.

The present study analysed a cohort of patients prospectively followed up after lens-sparing PPV for RRD. We used objective criteria to determine when cataract extraction should be indicated.

## Patients and methods

This prospective study was conducted at the Ophthalmology Unit of the University Hospital of Parma (Parma, Italy) and included patients undergoing PPV for RRD with preservation of the lens. The cohort included patients enrolled in a previous trial aimed to compare the primary reattachment rate in RRD cases treated by PPV combined with cataract extraction vs. PPV alone^5^. Subjects assigned to the lens-sparing group were offered the continuation of the follow up to check cataract progression. The original protocol had strict inclusion criteria with reference to patient age, the number and localisation of the retinal teras, the type of tamponade. The study was approved by the Area Vasta Emilia Nord Ethics Committee (protocol numbers 569/2018 and 7489/2019) and adhered to the tenets of the Declaration of Helsinki. Written informed consent was obtained from all participants. The inclusion criteria were an age of 18–65 years and prior lens-sparing PPV for RRD, with a lens opacity not exceeding the first grade for each category of the Lens Opacities Classification System III (LOCS III)^6^. The characteristics of the original RRD respected the inclusion criteria of the previous trial, i.e., up to three separate, superior retinal tears with an overall extension of retinal breaks < 90°; PVR not exceeding the grade B according to the updated classification of 1991. The exclusion criteria were any intraoperative complication involving the lens during PPV and postoperative complications, such as endophthalmitis or recurrence of RRD. In addition, diabetic retinopathy, inherited or age-related maculopathy or optic nerve disease.

The participants were examined 3, 6 and 12 months after PPV, and then on patient request due to perceived visual impairment or at the end of the follow-up period (May 2021). Each evaluation included assessment of the best-corrected visual acuity (BCVA) using Early Treatment for Diabetic Retinopathy Study (ETDRS) charts, intraocular pressure (IOP; by applanation tonometry), slit lamp examination under mydriasis (for lens opacity grading according to the LOCS III), macular optical coherence tomography (OCT) under mydriasis, and fundoscopy.

Follow-up ended when cataract extraction surgery (CES), performed by two surgeons (PM and ST), was clinically indicated. CES was indicated when the BCVA decreased by ≥ 15 letters on the ETDRS charts relative to the number at the 3-month follow-up visit, or when the spherical equivalent (SE) varied by ± 2.5 D compared to that measured 3 months after PPV. The primary study outcome was the time between PPV and the indication for CES. Secondary outcomes were the grade of cataract that developed during follow-up according to the LOCS III, and any correlations of CES with gender, age at the time of PPV, the calliper used (23–25-gauge), type of tamponade, macular status (on/off), axial length (AL) of the eye (evaluated on a flattened retina using the IOL Master instrument; Carl Zeiss Meditec) and the median IOP (of all values measured during follow-up). CES was performed using a standard phacoemulsification technique.

### Statistical analysis

The normality of the data distribution was checked using the Kolmogorov–Smirnov test. The mean and standard deviation (SD) were calculated for continuous variables with a normal distribution, and the median with interquartile range (IQR) for continuous variables with a non-normal distribution. The Friedman test was used for analysing ordinal variables. Factors potentially related to the indication for CES were examined by Kaplan–Meier survival analysis and the log-rank test. The univariate followed by multivariate Cox proportional hazards model was used to evaluate the association between the development of indication for CES (i.e. the study event), according to the above-mentioned refractive criteria, and all the possible influencing variables inferred on a clinical and scientifical basis. For each of the considered parameters the hazard ratio (HR) with 95% confidence interval (CI) was calculated. The significance threshold was set at p < 0.05. All analyses were performed using SPSS software (version 27.0; SPSS Inc.).

## Results

Thirty-six eyes of 36 patients (24 males, 12 females) were included in the study. Patient demographics and baseline ocular findings are presented in Table 1. Tamponade was performed after PPV with octafluoropropane (C_3_F_8_, 18%) in 33 eyes, and with polydimethylsiloxane (PDMS-1000) in 3 eyes. In these eyes the silicon oil was removed within 120 days from the first PPV. During the study period, 19 eyes (53%) developed the indication for CES, anyone before 6 months of follow up, and underwent cataract surgery. Considering all the included eyes, the median time from PPV to CES indication was 14.5 months (IQR: 12.0–24.8 months) and the mean preoperative BCVA was 0.62 ± 0.29 logMAR. At the last routinary post-operative visit, performed 4 to 6 weeks after phacoemulsification, the pseudophakic eyes had a mean BCVA of 0.19 ± 0.18 logMAR (p < 0.001 vs. preoperative). Mean BCVA was 0.26 ± 0.16 logMAR with mean SE of − 4.6 D at the baseline; 0.29 ± 0.18 logMAR with mean SE of − 5.0 D at the 6-month assessment. At the “end of the follow up” assessment the mean BCVA was 0.45 ± 0.27 logMAR and the SE − 7.8 D. Considering the eyes with gas tamponade separately from those which received the PDMS, the indication for CES involved 17 eyes (52%) in the former group and 2 eyes (67%) in the latter one. The median time from PPV to CES indication was 19.0 months (IQR: 5.0–25.0 months) for the gas tamponade eyes and 8.8 months (IQR: 4.5–13.0 months) for the eyes with PDMS. The variable “tamponade” was included in the multivariate analysis as detailed below.

**Table 1:** Patients’ demographics and ocular findings at baseline.

M/F	24/12
Age (years)	51.8 ± 9.5
Eye (RE/LE)	20/16
AL (mm)	26.09 ± 2.09
BCVA (LogMAR)	0.62 ± 0.29
SE (D)	− 3.93 ± 4.97
IOP Average (mmHg)	16 ± 2.8
Macular involvement (ON/OFF)	15/21
GAUGE (23/25)	28/8
PPV duration (minutes)	79 ± 24.5
END follow up (months)	20 ± 13

*M* males, *F* females, *AL* axial length, *BCVA* best corrected visual acuity, *SE* spherical equivalent, *IOP* intraocular pressure, *PPV* pars plana vitrectomy.

**Figure 1:**
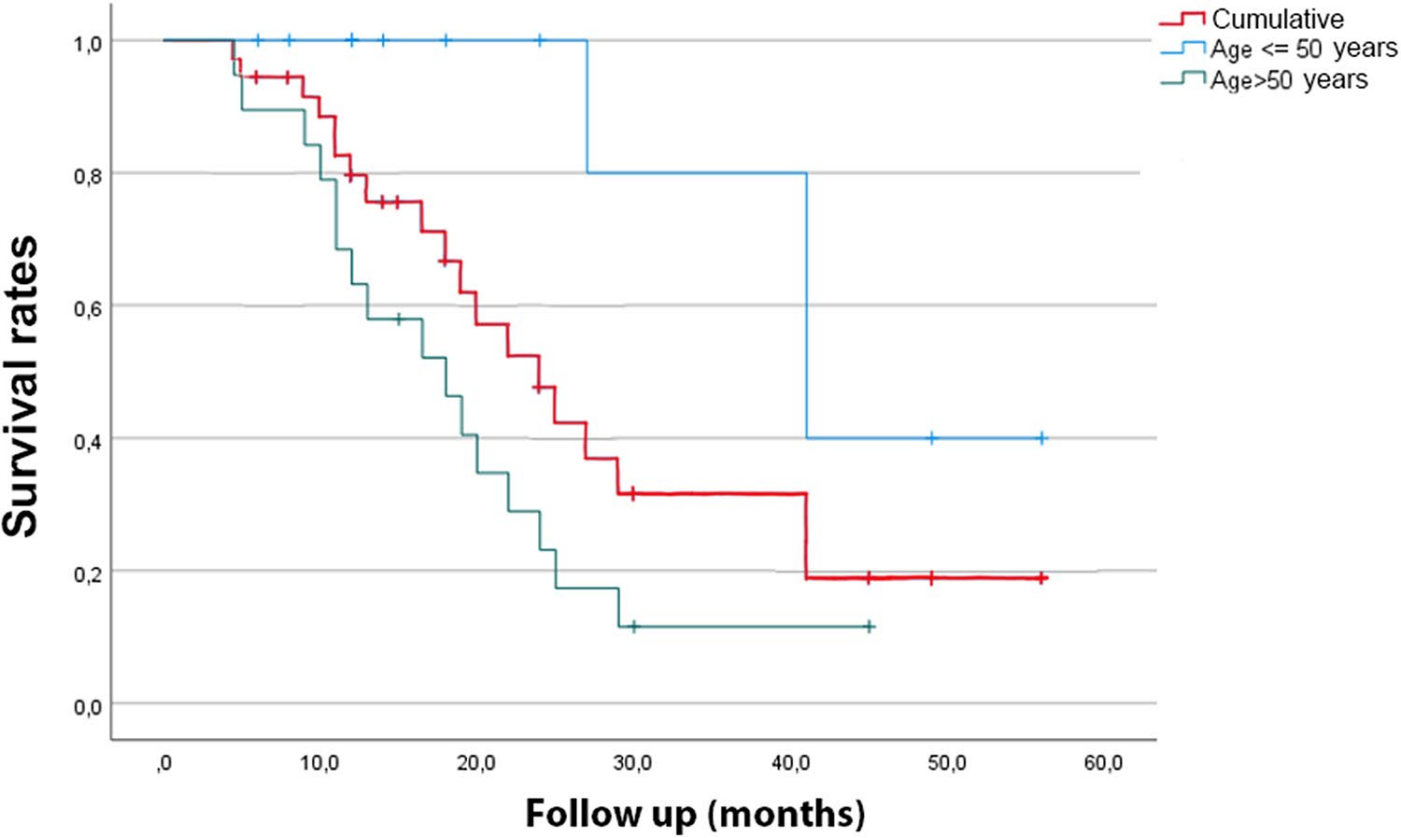
Timing of events, in terms of indication for cataract extraction surgery, over the follow-up period. The figure includes the cumulative curve, and the curves referring to patients aged ≤ 50 and > 50 years.

The distribution of events over the follow-up period is shown in Fig. 1. The figure represents either the cumulative survival curve, or the curves referred to the study population subdivided in subjects aged ≤ 50 years and > 50 years at the time of PPV. The difference between the event rate in the two subgroups was significant (p < 0.01). As complications of subsequent phacoemulsification surgeries, one case of capsule rupture and one of sectorial zonular dialysis occurred. In nine among the operated eyes (42%), including those with intraoperative complications, the surgeons observed wide fluctuations of the anterior chamber depth, which led to marked lowering of the infusion bottle and injection of larger amounts of viscoelastic material into the eye.

**Figure 2:**
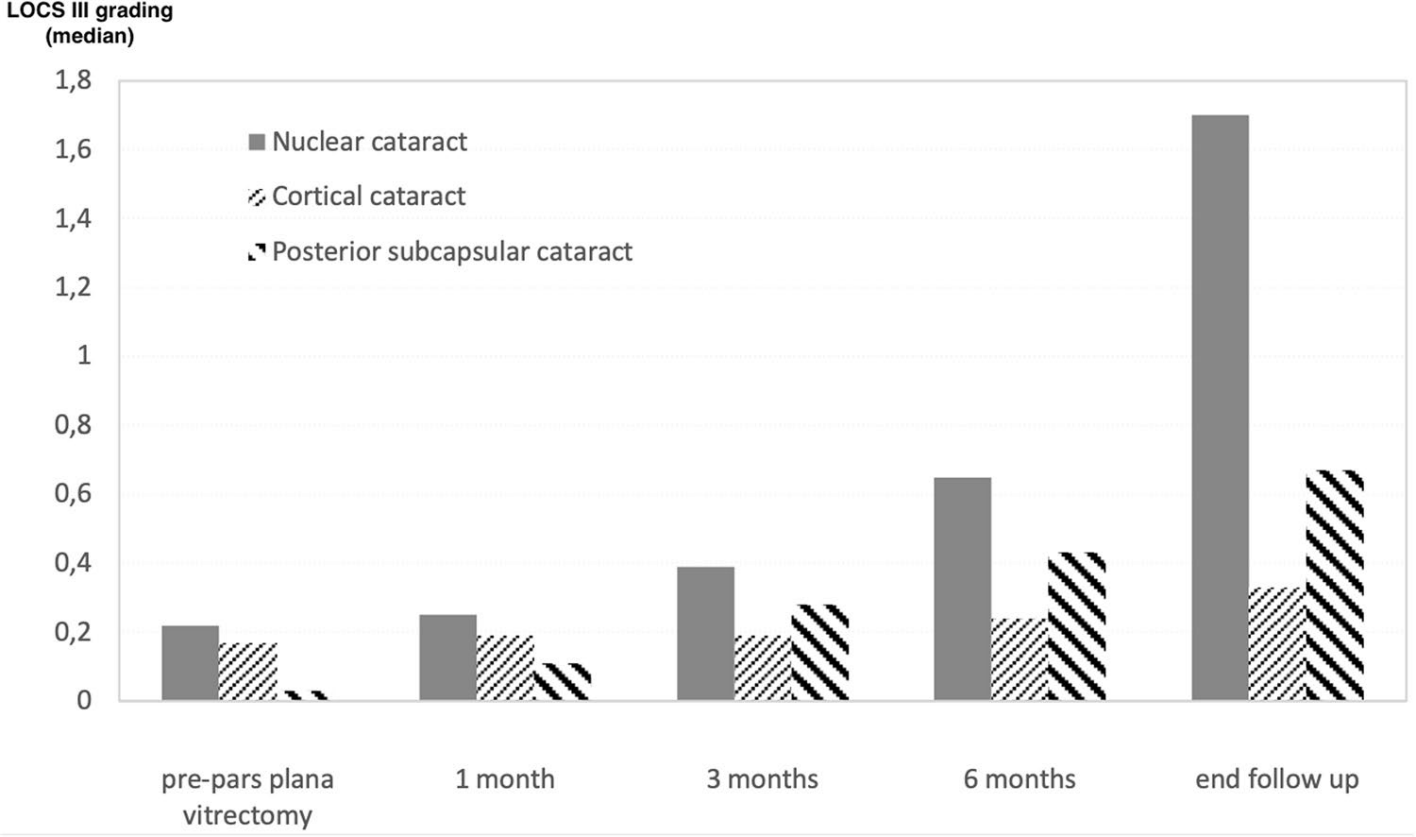
Progression of the various types of cataract according to the LOCS III grading system. The 12-month timepoint was omitted in order not to miss the cases indicated for surgery between the 6-month and the 12-month evaluation.

Progression of the various types of cataract, according to the LOCS III, is shown in Fig. 2. Relative to the pre-PPV levels, cataract progressed significantly starting from 6 months after PPV for the N and PSC forms (p < 0.001 and p < 0.005, respectively). For these two types, a further significant increase was noted between 6 months after PPV and the end of the follow-up period (p < 0.001 and p = 0.010, respectively). No such variation was observed for the cortical (C) form over the follow-up period (Table 2). The OCT evaluation excluded the presence of tractional macular edema in all the study eyes.

**Table 2:** Cataract development for each LOCS III type over the main follow up timepoints.

	PRE-PPV VS. 1 month	PRE-PPV VS. 3 months	PRE-PPV VS. 6 months	PRE-PPV VS. END FU	6 months VS. END FU
N	0.320	0.083	< 0.000*	< 0.000*	< 0.000*
C	0.320	0.324	0.083	0.056	0.373
P	0.080	0.018	0.005*	< 0.000*	0.010

*PPV* pars plana vitrectomy, *FU* follow up, *N* nuclear cataract, *C* cortical cataract, *P* posterior subcapsular cataract.

*statistical significance achieved.

The association of indication for CES with sex (as internal control for fitness), age at PPV, eye axial length, pre-PPV macular status (ON/OFF), PPV duration, type of tamponade (gas or silicone oil), and mean intraocular pressure during the follow up post-PPV were tested and shown in Table 3 including the HR and the 95% CI. In univariate Cox regression analysis only the patient age at the time of PPV showed significant association with cataract development (p = 0.011). In multivariate analysis, two more prognostic factors for earlier CES indication achieved the threshold of significance: (a) again the patient age at the time of PPV (p = 0.002); (b) the tamponade, with reference to the PDMS (p = 0.013); (c) the macular involvement by the RRD (p = 0.043). In particular, the HR for CES indication increased 1.173 times with every additional year of age.

**Table 3:** Univariate (upper part) and multivariate (lower part) COX regression analyses referred to the development of the indication for cataract extraction surgery.

	HR	95% CI	*p* value
**COX univariate**			
Mean post-PPV IOP (mmHg) ((mmHg)AVER	1.059	0.864–1.297	0.580
PPV duration (minutes)	1.002	0.984–1.021	0,801
Axial length (mm)	1.070	0.835–1.370	0.595
SEX (referred to male gender)	1.670	0.665–4.196	0.275
Age at PPV (years)	1.079	1.017–1.144	0.011*
Pre-PPV macula status (referred to OFF) OFF)	0.675	0.274–.666	0.394
PPV tamponade (referred to PMDS)	0.297	0.063–1.463	0.125
**COX multivariate**			
Mean post-PPV IOP (mmHg) ((mmHg)AVER	1.193	0.902–1.579	0.216
PPV duration (minutes)	1.013	0.989–1.038	0,298
Axial length (mm)	1.346	0,963–1.882	0.082
SEX (referred to male gender)	0.921	0.324–2.620	0.877
Age at PPV (years)	1.173	1.062–1.295	0.002*
Pre-PPV macula status (referred to OFF) OFF)	0.261	0.071–0.958	0.043*
PPV tamponade (referred to PMDS)	0.065	0.008–0.567	0.013*

## Discussion

The present study prospectively analysed cataract progression, using the LOCS III grading system, in a cohort of eyes treated with PPV for RRD. The potential influence of various demographic, clinical and surgical characteristics was also evaluated. Factors related to cataract progression after vitrectomy have been extensively discussed in the literature. To date, most studies have been retrospective, including large samples but with low homogeneity in terms of the study populations, timing of follow-up visits, surgeons and investigators^7,8^. The present study was unique in calculating the time to CES indication following PPV. The novelty is chiefly methodological, consisting in the “ex ante” definition of the level of lens opacification and/or refractive variation required to indicate CES. This way to collect data on the parameters possibly influencing cataract development ensures minimal biases of correlation. The median time between PPV and CES (14.5 months) was similar to that in a prospective series^9^, but longer than the means reported in other studies examining similar characteristics, such as small-gauge PPV or RRD treated with gas tamponade^7,10,11^. Regarding the type of cataract, the N form progressed significantly according to the LOCS III starting from 6 months after PPV, as did the PSC type. In contrast, the C type showed no significant progression during the whole follow-up period, in line with previous observations^10–13^. Progression of the N and PSC forms supported the concept that noxa mainly affected the posterior surface of the lens. These forms may result from increased exposure to oxygen via the retinal vasculature due to an absence of vitreous gel, prolonged liquid flow from the pars plana and the use of steroids as vitreal stainer during vitrectomy^14,15^. Unlike the N and SCP forms, C cataracts are associated with extensive disruption of cell structure beginning near the equator of the lens^16^.

Three parameters in this study were significantly correlated with cataract progression: patient age, the use of PDMS tamponade and the macular involvement by the original RRD. The correlation with the patient age at the time of the PPV was already evident in the univariate analysis, and in line with former or more recent literature reports^17,18^. In older patients (at the time of PPV), the CES indication was earlier, with the hazard ratio approximately doubling for every 5-year increase in age. This supports the preference for lens-sparing PPV to treat RRD in younger patients, considering also the increased technical difficulty of phacoemulsification in vitrectomized eyes. Certainly, the lens-sparing technique implies some differences as compared to the PPV combined with cataract extraction (i.e. phacovitrectomy, PCV). These essentially concern the enhanced retinal visualization during posterior segment surgery; the better access to the vitreous base allowing for a more extensive vitrectomy and endolaser treatment (thereby ensuring more extensive gas filling and better tamponade of retinal breaks) when the lens removal occurs before the PPV. However, just the prospective trial connected to the present study reported that the preservation of the crystalline lens at the time of PPV ensured similar outcomes to those obtained with PCV, in terms of the retinal reattachment rate and safety during postoperative management^5^.

The correlation between cataract development and presence of PDMS identified in the present study was in line with the literature^7,11^. The statistically significant results obtained in the small number of eyes with PDMS in this study confirms the appropriateness of the design. However, the further surgery required to remove the silicon oil could have biased the outcome for the indication of CES. On the one hand, the fluid exchanges in the vitreous cavity may promote cataract progression; on the other hand, the removal of silicone oil is known to improve retinal sensitivity and, to some extent, visual acuity^19^.

The evidence that the macula OFF status is related to earlier indication for CES is difficult to explain logically since multiple factors can influence the assessment of visual acuity in these eyes. The visual acuity can potentially continue to improve even beyond 3 months once macula has been reattached. Depending on the level of vision achieved at 3 months post RRD repair, further degradation in visual acuity may vary for a similar progression of cataract. The lack of certain explanation and the borderline statistical significance make this point worthy of further investigations.

The limited sample size represents the major limitation of this study. On the other hand, the study could have benefitted from the criteria which limited the enrolment. These criteria contributed to a highly homogeneous cohort in terms of pre-surgery disease, the technical approach and post-PPV follow-up duration. Through prospective observation, visual and refractive conditions that indicate CES were clearly identified and located in time.

## Data Availability

All data and material are available from the corresponding author.
